# Piceatannol-Loaded Bilosome-Stabilized Zein Protein Exhibits Enhanced Cytostatic and Apoptotic Activities in Lung Cancer Cells

**DOI:** 10.3390/pharmaceutics13050638

**Published:** 2021-04-29

**Authors:** Nabil A. Alhakamy, Giuseppe Caruso, Mohammed W. Al-Rabia, Shaimaa M. Badr-Eldin, Hibah M. Aldawsari, Hani Z. Asfour, Samah Alshehri, Sami H. Alzaharani, Meshari M. Alhamdan, Waleed Y. Rizg, Ahmed N. Allam

**Affiliations:** 1 Department of Pharmaceutics, Faculty of Pharmacy, King Abdulaziz University, Jeddah 21589, Saudi Arabia; nalhakamy@kau.edu.sa (N.A.A.); haldosari@kau.edu.sa (H.M.A.); wrizq@kau.edu.sa (W.Y.R.); 2 Advanced Drug Delivery Research Group, Faculty of Pharmacy, King Abdulaziz University, Jeddah 21589, Saudi Arabia; 3 Center of Excellence for Drug Research and Pharmaceutical Industries, King Abdulaziz University, Jeddah 21589, Saudi Arabia; 4 Department of Drug and Health Sciences, University of Catania, 95125 Catania, Italy; giuseppe.caruso2@unict.it; 5 Department of Medical Microbiology and Parasitology, Faculty of Medicine, King Abdulaziz University, Jeddah 21589, Saudi Arabia; mwalrabia@kau.edu.sa (M.W.A.-R.); hasfour@kau.edu.sa (H.Z.A.); 6 Department of Pharmaceutics and Industrial Pharmacy, Faculty of Pharmacy, Cairo University, Cairo 11562, Egypt; 7 Department of Pharmacy Practice, Faculty of Pharmacy, King Abdulaziz University, Jeddah 21589, Saudi Arabia; salshehri1@kau.edu.sa; 8 Family Medicine Department, Faculty of Medicine, King Abdulaziz University, Jeddah 21589, Saudi Arabia; shaalzahrni4@kau.edu.sa (S.H.A.); malhamdan1@kau.edu.sa (M.M.A.); 9 Department of Pharmaceutics, Faculty of Pharmacy, Alexandria University, Alexandria 21521, Egypt

**Keywords:** piceatannol, bilosomes, zein, A549cells, apoptosis

## Abstract

Piceatannol (PIC) is a naturally occurring polyphenolic stilbene, and it has pleiotropic pharmacological properties. Moreover, PIC has cytotoxic actions among various cancer cells. In this work, preparations of PIC-loaded bilosome–zein (PIC-BZ) were designed, formulated, and characterized, and the optimized PIC-BZ cytotoxic activities, measured as half maximal inhibitory concentration (IC_50_), against lung cancer cell line was investigated. Box–Behnken design was utilized in order to examine the effect of preparation factors on drug entrapment and particle size. PIC-BZ showed a spherical shape after optimization, and its particle size was determined as 157.45 ± 1.62 nm. Moreover, the efficiency of drug entrapment was found as 93.14 ± 2.15%. The cytotoxic activity evaluation revealed that the adjusted formulation, which is PIC-BZ formula, showed a substantially smaller IC_50_ versus A549 cells. Cell cycle analysis showed accumulation of cells in the G2-M phase. Moreover, it showed in the sub-G1 phase, a rise of cell fraction suggestion apoptotic improving activity. Increased early and late phases of apoptosis were demonstrated by staining of cells with annexin V. Furthermore, the cellular caspase-3 protein expression was significantly raised by PIC-BZ. In addition, the wound healing experiment confirmed the results. To conclude, compared to pure PIC, PIC-BZ demonstrated a higher cell death-inducing activity against A549 cells.

## 1. Introduction

Lung cancer is a malignant lung tumor characterized by unregulated proliferation of lung tissue body cells. The tumor cells growth, by the process of metastasis, can spread outside the lung into nearby tissues or other body areas. The majority of cancers that start in the lung are carcinomas [1]. The standard treatment strategies for this disorder are chemotherapy and/or surgery. Natural products are receiving significant interest as chemotherapeutic agents for cancer [1,2]. A broad variety of chemical groups of plant active ingredients have been investigated for their potential role in lung cancer treatment [3,4,5]. The active ingredients include polyphenols and alkaloids [6]. A phenolic compound named piceatannol (PIC) belongs to the stilbenoids class. PIC is a resveratrol metabolite with many pharmacological effects, including anti-tumor activity. There have been studies of PIC cytotoxicity against lymphoma, melanoma, leukemia, and colon and prostate cancer cells [7]. Furthermore, PIC synergizes cancer chemotherapeutics with anti-cancer activities to overcome drug resistance [8]. Apoptosis protein suppression, mitochondrial fission, and gene regulation are thought to induce this activity. There are other reports that indicate stilbenes act as apoptosis inducers and multidrug resistance modulators [9]. In cancer management, the anti-cancer activity of PIC is promising. As an explanation for this finding, promotion of apoptosis through upregulation of mRNA expression is provided [10].

Nanotechnology has shown a leading role in enhancing chemotherapeutic drugs for cancer. It is essential to note that PIC albumin nanoparticles have been reported to be effective in a murine colon cancer model [11]. Although all kinds of nanostructures are stated to be utilized as anti-cancer agents, there are some specific advantages of lipid-based systems. Because of their lipophilic nature, their ability to penetrate the biological-barriers is more effective than the nano-structure polymer [12]. In the pharmaceutical field, lipid-based vesicular systems have been extensively studied as carriers in order to improve drug bioavailability and/or facilitate their targeting to certain tumors or organs selectively [13]. The first generation of such systems is called liposomes. To further enhance the drug stability and delivery features of such systems, modified vesicular systems, which are structurally similar to liposomes, have recently appeared. The use of nano-colloidal drug delivery systems is among the numerous techniques used to improve drug–receptor interaction. Researchers are introducing certain techniques to deal with impediments associated with the difficulties of supplying individual target tissues with the needed therapeutic moieties. Vesicular carriers are one form of nano-colloidal system that has gained a large amount of attention for distributing poorly soluble drugs and proteins/peptides [14,15,16,17]. There are various structures in vesicular carriers, including unilamellar or multilamellar spherical structures, often consisting of lipid molecules organized into orientation bilayers and capable of encapsulating drug molecules [18,19,20]. The capacity of traditional vesicular carriers such as niosomes and liposomes to increase the oral bioavailability of therapeutic agents has been shown. However, since traditional vesicles’ efficacy has been hampered by their gastrointestinal track instability, it was important to modify their bilayer constructs in order to increase their in vivo efficiency [20,21].

Lately, several research efforts have demonstrated the feasibility of introducing bile salts into bilayer vesicles in order to increase their in vivo tolerance and efficiency after oral route administration [22,23,24,25]. Bile salts, which are endogenous detergents, are applied as permeability enhancers in drug delivery. Therefore, they facilitate the penetration of active pharmaceutical ingredients (APIs) across biological barriers, including the blood–brain barrier, intestinal wall, skin, cornea, and the nasal mucosa. Bilosomes, which are bile salts containing niosomes, have been utilized to improve the oral bioavailability of different medications and macromolecules [26,27,28,29,30]. They include closed bilayer vesicles of non-ionic amphiphiles with incorporating bile salts. In previous research, the potential of utilizing bilosomes in order to allow successful drug delivery has been confirmed [26,27,28,29,30]. Bilosomes are more resistant against gastrointestinal fluids, such as enzyme and bile salts, due to the presence of bile salts in the lipid bilayers, thereby protecting the entrapped APIs [31,32,33]. The use of negatively charged bile salts (such as sodium deoxycholate) improves the vesicular system’s stability [34,35].

Zein is a prolamine class maize protein that was first isolated in 1821 [36]. The characteristics of zein broadened its application as a coating and encapsulating material in both food and pharmaceutical industries [37,38,39,40,41,42,43,44]. The U.S. Food and Drug Administration classified zein as Generally Recognized as Safe (GRAS) material [45].

The aim of this work was to evaluate the efficacy of PIC-loaded bilosome–zein (BZ) nanoparticles in suppressing A549 lung cancer cells. Response surface design was utilized for the formulation of PIC-BZ. The optimized PIC-BZ was characterized for vesicle size entrapment and PIC release. Moreover, the optimized PIC-BZ formula was evaluated in terms of IC_50_, pro-apoptotic activity, and wound healing inhibition in A549 lung cancer cells.

## 2. Materials and Methods

### 2.1. Materials

PIC, cholesterol, zein, sodium deoxycholate, chloroform, methanol, and acetonitrile were obtained from Sigma-Aldrich Inc. (St. Louis, MI, USA). A549 cells were obtained from NCCS, Pune, India. All the other chemicals and reagents were of the analytical grade.

### 2.2. Experimental Design

Response surface design, specifically Box–Behnken, was utilized in order to prepare PIC-BZ using version 12 of Design-Expert software (Stat-Ease Inc., Minneapolis, MN, USA). There are 3 separate variables, namely, cholesterol: Span 20 molar ratio (X_1_), bile salt molar concentration (X_2_, mM), and zein concentration (X_3_, %*w*/*w*) that were studied for their effects on the response and particle size (Y). Table 1 shows the levels of the investigated factors. As per the design, 17 experimental trials were generated by the software, including 5 center points.

Table 2 displays the overall calculated particle size for each trial as well as the cumulative levels of the factors for the study runs. The best-fitting sequential model was chosen on the basis of the model fit statistical results. The selection was performed on the basis of the greatest adjusted and predicted R^2^ and the least predicted residual sum of squares (PRESS). 2D contour plots were utilized to display the effect of the factors and the interaction between them.

#### 2.2.1. Preparation of PIC-BZ

A modified thin-film hydration procedure was used in order to prepare PIC-BZ, as previously described [29,46]. Briefly, PIC (20 mg), cholesterol, and Span 85 (amount as indicated in the design of the experiment) were dissolved in 10 mL chloroform. A specified amount of zein, according to the design, was dissolved in methanol and then mixed with the chloroform solution in a round bottom flask. Under reduced pressure at 65 °C, the organic solution was evaporated using a rotary evaporator until a thin and dry film was formed. Then, the formed film was kept at 25 °C in a vacuum oven for 24 h to confirm the full removal of the residues of organic solvent. Sodium deoxycholate was dissolved in double-distilled water (10 mL) and hydrated the dried film under rotation for 1 h. The formed PIC-BZ dispersion was subjected to sonication for 5 min (water bath sonicator at 25 °C), followed by probe sonication for 60 s, then stored at 4 °C until use.

#### 2.2.2. Measurement of Particle Size

PIC-BZ vesicle size was determined by appropriate dilution in double-distilled water (1:10) using a Zetasizer Nano ZSP particle size analyzer instrument (Malvern, UK).

#### 2.2.3. Optimization of PIC-BZ

The examined PIC-BZ preparation parameters were optimized utilizing a numerical approach subsequent desirability technique. The optimization process aimed at minimizing the size of the prepared PIC-BZ. The predicted optimized formulation was prepared for further characterization.

### 2.3. Characterization of Optimized Formulation

#### 2.3.1. PIC-BZ Entrapment Determination

Analysis of the API content of emulsomes relative to the overall concentration added was used to evaluate the efficacy of entrapment of PIC in emulsomes. As a result, *n-*propanol (50 percent *v/v*) in PBS was used to disrupt a pre-weighted component of the emulsomes (pH 7.4).

The prepared PIC-BZ was exposed to centrifugation for 45 min at 30,000 rpm (2 cycles). After that, a pre-weighed amount of lyophilized PIC-BZ was exposed to disruption with *n-*propanol (50% *v/v*) in phosphate-buffered saline (PBS) at pH 7.4 and then subjected to HPLC analysis (Agilent 1200, Agilent Technologies, Santa Clara, CA, USA) [47]. The entrapped PIC% was calculated using Equation (1).

(1) $$\mathrm{Entrapped}\;\mathrm{PIC}\;\left(\%\right)=\frac{\mathrm{amount}\;\mathrm{of}\;\mathrm{PIC}\;\mathrm{in}\;\mathrm{BZ}}{\mathrm{amount}\;\mathrm{of}\;\mathrm{PIC}\;\mathrm{used}}\times 100\;$$

#### 2.3.2. PIC release from the Optimized PIC-BZ Formula

The PIC in vitro release from the BZ was calculated after a stated method [11,48]. The release analysis was carried out with 0.1 M PBS at pH 7.4 containing Tween 80 (0.1%). A sufficiently pre-weighed volume of PIC-BZ containing 2 mg was attached to a previously activated dialysis bag (MWCO = 12,000 Da). The temperature of the sample placed in the dialysis bag was set at 37 °C in a water bath shaker. Then, several samples were withdrawn at different periods, including 0.5, 1, 2, 4, 6, 8, 10, 12, 18, and 24 h. After that, PIC amount released was analyzed by utilizing an HPLC method as previously reported [47]. This experiment was performed in triplicate.

#### 2.3.3. Determination of IC_50_ Values

In this part, the process involved seeding of A549 cells in Glutamax containing DMEM/F12 medium (Gibco-Life Technologies, Rockville, MD, USA). It was also supplemented with 10 mM HEPES buffer, fetal bovine serum (10%), penicillin G sodium (100 units/mL), and streptomycin sulfate (100 μg/mL). The prepared cells were then incubated with various concentrations of plain-BZ, PIC, or PIC-BZ (dispersed in the cell culture medium) in a CO_2_ incubator at logarithmic intervals of 48 h at 37 °C. In order to determine the IC_50_ values, we employed a MTT assay method as previously described [49].

#### 2.3.4. Cellular Uptake Analysis of Optimized PIC-BZ

The A549 cells were incubated overnight (1 × 105 cells per dish). The cells were incubated at 37 °C in the presence of 5% CO_2_ for 2 and 4 h after being treated with 5.3 µM PIC-BZ, as well as equivalent concentrations of PIC. The monolayers were washed three times with PBS, and then a lysis solution (PBS containing 0.025% trypsin and 1% Tween 20) was added for 30 min at 37 °C. Aliquots of the cell lysates were collected and analyzed by HPLC [47].

#### 2.3.5. Cell Cycle Analysis

The cell cycle DNA distribution was determined using a FACS Calibur flow cytometer instrument (BD Bioscience, San Jose, CA, USA) as previously described [50,51,52]. In brief, many plates (6-well cell culture) were utilized and seeded with about 5 × 10^3^ A549 cells per well. Subsequently, 0.1 µM PIC-BZ was added to the seeded cells. After that, for 24 h, an equivalent amount of Blank-BZ, PIC-raw, and PIC-BZ were added. After collection and washing, a DNA Reagent Kit CycleTEST PLUS (Becton Dickinson Immunocytometry Systems, San Jose, CA, USA) was utilized for cell cycle analysis. In comparison to peripheral blood mononuclear cells (PBMCs) with a fixed content of DNA, the DI (DNA Index) of the samples was calculated. The DI was determined from the mean of G0/G1 peaks of the sample and the control (PBMC) populations. Propidium iodide (PI) was used for staining. A minimum of 20,000 events were acquired for each treatment. Finally, in order to study the cell cycle distribution, we used a software named CELLQUEST (Becton Dickinson Immunocytometry Systems, San Jose, CA, USA).

#### 2.3.6. Annexin V Assay

To assess apoptosis, we performed a dual staining technique. A plate that contained 6 wells was utilized with a cell density of 5 × 10^3^ cells per well to incubate A549 cells with blank-BZ, PIC-raw, and PIC-BZ with relation to 0.1 µM PIC. In addition, a control sample that contained untreated cell was incubated in this work. A kit from BD Bioscience (San Jose, CA, USA) was utilized for staining of cells. After 24 h of incubation, the examined cells were collected by centrifugation. During the next step, they were re-suspended in 500 μL of 1× binding buffer. After that, 5 μL of each PI (BD Bioscience) and annexin V-FITC were added and incubated in the dark for 5 min at room temperature. In this experiment, a FACS Calibur flow cytometry instrument (BD Bioscience) was used for the analysis. Moreover, Phoenix Flow Systems multicycle software (San Diego, CA, USA) was utilized to analyze the results.

#### 2.3.7. Caspase-3 Assay

A commercially available ABCAM kit (Cambridge, UK) was used for the quantification of caspase-3. The prepared samples with the A549 cells (5 × 10^3^ cells per well) were incubated. Then, the cells were washed and lysed. Finally, as suggested by the manufacturer of the kit, each cell lysate was treated, and the absorbance was evaluated in order to measure the concentration of caspase-3 at 405 nm. It was stated as pg/mg protein determined utilizing a kit (BCA protein assay) (Sigma-Aldrich, St. Louis, MI, USA) [35,53].

#### 2.3.8. Wound Scratch Assay

This method was carried out in order to measure cell proliferation under our experimental conditions. Approximately 0.6 million cells were seeded in plates that had 35 mm in diameter. Cells were treated the next day for 1 h with 2 μg/mL mitomycin C. Then, the cells available in the middle of the plates were scraped by sterile tip in order to form a wound. Blank-BZ, PIC-raw, and PIC-BZ sub-toxic doses were applied to the plates, and each plate was incubated for 24 h. Axio Vision Rel 4.8 imaging software (Carl Zeiss Microimaging GmbH; Göttingen, Germany) was used to assess the wound’s depth. The result obtained was plotted as a percentage of wound closure relative to power. The controls were untreated samples, and 100% were deemed to be covered.

#### 2.3.9. Statistical Analysis

In this work, data are shown as mean ± SD. IBM SPSS statistics software, version 25 (SPSS Inc., Chicago, IL, USA), was used for statistical analysis. The one-way analysis of variance (ANOVA) test was used to compare the means. Then, the Tukey test was performed as a post hoc test. The *p*-values < 0.05 were considered significant.

## 3. Results

### 3.1. Experimental Design

#### 3.1.1. Fit and Diagnostic Statistics

Among the investigated sequential models, the best fitting model for PIC-BZ particle size was the quadratic model as per its highest correlation (R^2^) and lowest predicted residual sum of square (PRESS) (Table 3).

**Figure 1:**
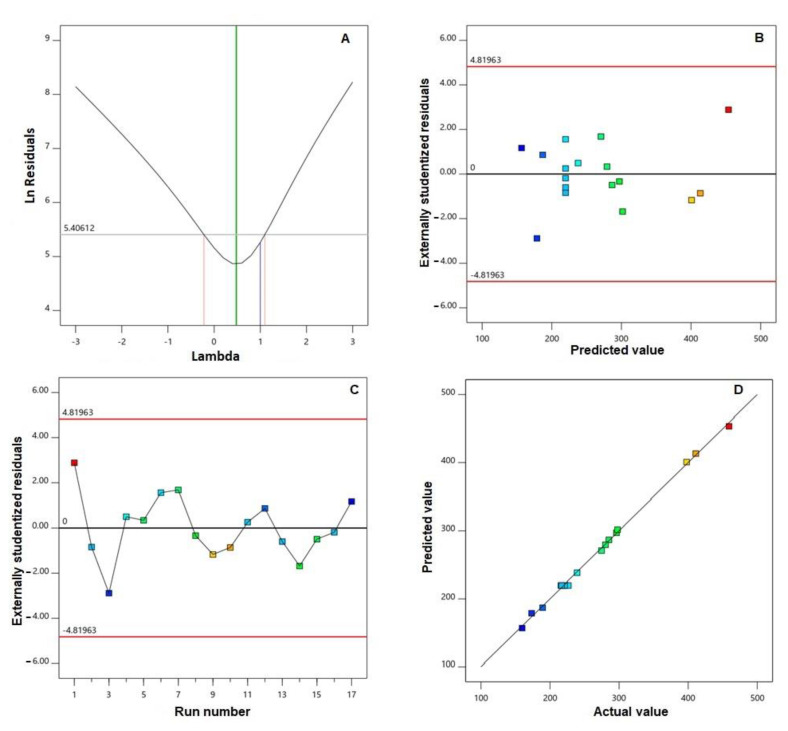
Diagnostic plots for globule size of PIC-BZ: (**A**) Box–Cox plot for power transforms, (**B**) externally studentized residuals vs. predicted values plot, (**C**) externally studentized residuals vs. run number plot, and (**D**) predicted vs. actual values plot.

The adequate precision value was 73.39, while the lack of fit F-value was 2.24 (*p* = 0.2259). Diagnostic plots for particle size, developed for checking the goodness of fit of the chosen model, are displayed in Figure 1.

The best lambda (λ) value (represented by the green line) in Box–Cox plot for power transforms (Figure 1A) was 0.48. The 95% confidence interval around this λ (shown as red lines) was −0.22 to 1.10. The externally studentized residuals vs. predicted response plot (Figure 1B) and the residual vs. run plot (Figure 1C) displayed randomly distributed points in between the limits. Furthermore, the predicted vs. actual particle size plot (Figure 1D) showed good linear pattern.

#### 3.1.2. Variable Influence on Particle Size (Y)

The size of particulate delivery systems is a crucial parameter affecting the release pattern and subsequent passage of active ingredients across the biological membranes. PIC-BZ showed nano-sized vesicles fluctuating from 159.6 ± 4.6 to 459.2 ± 16.3 nm (Table 2). The importance of the quadratic model was verified by analysis of variance (ANOVA) for particle size, with an F-value of 465.88 (*p* = 0.0001). In terms of coded factor, the Equation (2) for the best fitting quadratic model was generated as follows:

Y = 219.84 − 73.25 X_1_ − 48.84 X_2_ + 64.36 X_3_ + 8.23 X_1 × 2_ − 14.03 X_1_X_3_ − 7.00 X_2_X_3_ + 29.86 X_1_^2^ + 20.78X_2_^2^ + 52.58 X_3_^2^ (2)

**Figure 2:**
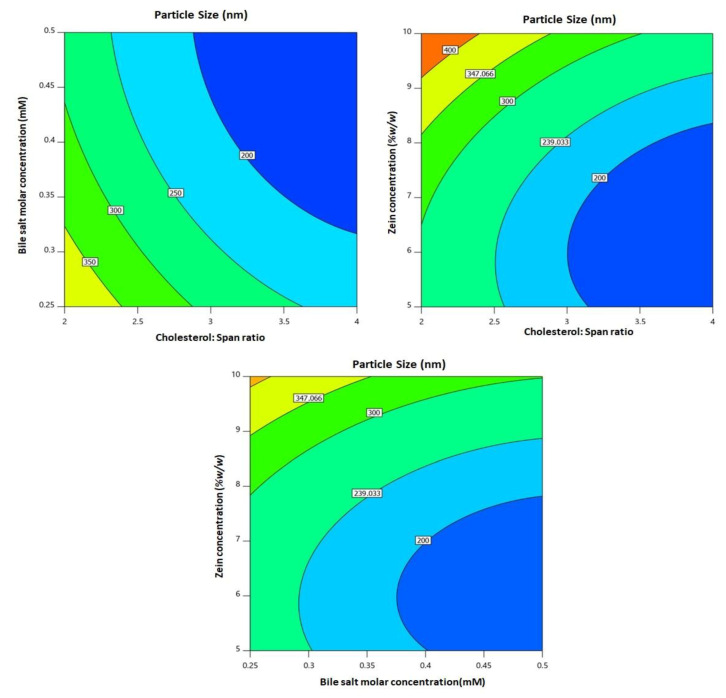
2D contour plot for the influence of formulation variables on the particle size of PIC-BZ.

All linear (X_1_, X_2_, and X_3_) and quadratic (X_1_^2^, X_2_^2^, and X_3_^2^) terms referring to the three studied variables had a major impact on particle size (*p* = 0.0001). In addition, the interaction terms were also significant (*p* = 0.0164, 0.0011, and 0.0032 for X_1_X_2_, X_1_X_3_, and X_2_X_3_, respectively). Figure 2 illustrates the two-dimensional contour plots for the effects and the interactions of the studied variables on particle size.

It can be seen that the particle size decreased at higher cholesterol/span ratio and bile salt molar concentrations, while it increased at higher zein concentrations.

#### 3.1.3. Optimization

**Figure 3:**
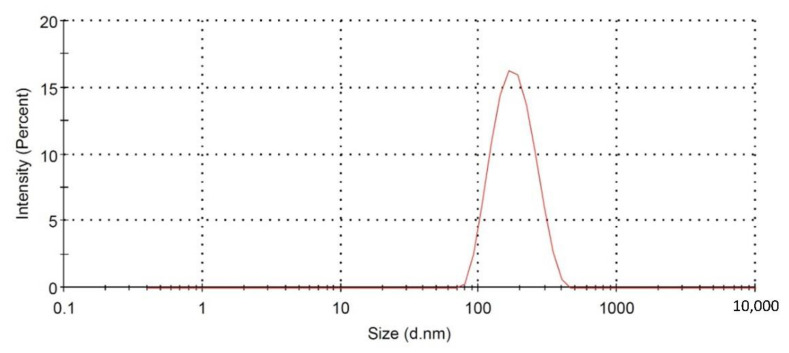
Particle size distribution of the optimized PIC-BZ.

Following the constraint previously set to particle size, the optimized levels of the formulation variables were anticipated with overall desirability of 0.981. The predicted levels were 1:3.977 for cholesterol/span ratio, 0.435 mM for bile salt concentration, and 7.052% *w*/*w* for zein concentration. The optimized formulation was prepared and evaluated for characteristics and biological activity in cancer cells. The percentage error (1.818%) between the predicted (155.13 nm) and observed particle sizes (157.27 nm, Figure 3) was relatively small, confirming the successfulness and validity of the optimization process.

### 3.2. Characterization and Evaluation of Optimized PIC-BZ Formulation

#### 3.2.1. PIC-BZ Entrapment Determination

The results of PIC entrapment efficiency in PIC-BZ showed high PIC entrapment within the nano-system of 93.14 ± 2.15%.

#### 3.2.2. PIC Release from the Optimized PIC-BZ Formula

**Figure 4:**
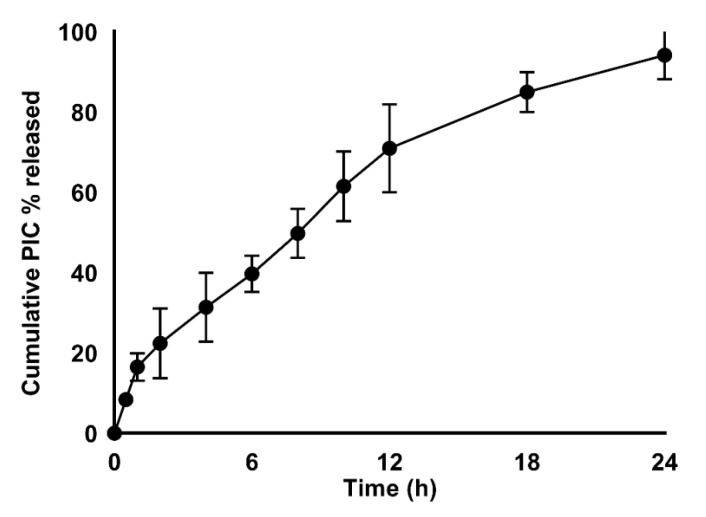
In vitro release profile of PIC-BZ in PBS (pH 7.4, 0.1 M) comprising 0.1% of Tween 80 at 37 ± 0.5 °C.

The in vitro dissolution of PIC from the BZ was studied. The drug release profile is illustrated in Figure 4.

The results showed that the prepared BZ provided a satisfactory gradual release profile. Moreover, at 8 h, it showed about 50% of drug release (49.8% ± 6.1). By 24 h, the drug release was about 94.2 ± 5.9%.

#### 3.2.3. Determination of IC_50_ Values

**Figure 5:**
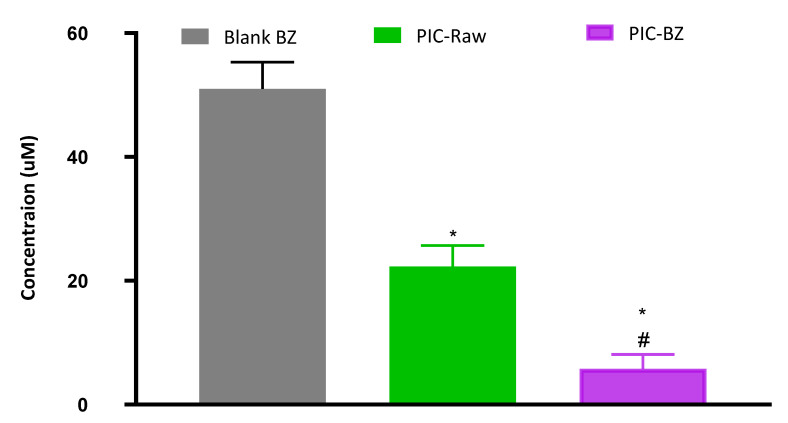
Cytotoxicity of PIC-BZ in A549. Cells were incubated with Blank-BZ, PIC-raw, or PIC-BZ for 48 h, and IC_50_ values were determined using MTT assay. Data are the mean of 4 independent experiments ± SD. * Significantly different from Blank-PZ, *p* < 0.05; # significantly different from PIC-raw, *p* < 0.05.

The MTT assay was used to evaluate the IC_50_ values in A549 cells. The results showed that IC_50_ values were significantly reduced for the PIC-BZ when compared to PIC-raw. The IC_50_ value for the PIC-BZ was 5.78 ± 2.3 µM, while it was 22.3 ± 3.4 µM for the PIC-raw and 51.4 ± 4.3 µM for Blank-BZ (Figure 5).

Both preparations (PIC-raw and PIC-BZ) had IC_50_ values greater than 30 μM against non-cancerous endothelial cells of EA.hy926, demonstrating a selective anti-cancer activity against cancerous cells.

#### 3.2.4. Cellular Uptake of Optimized PIC-BZ

**Figure 6:**
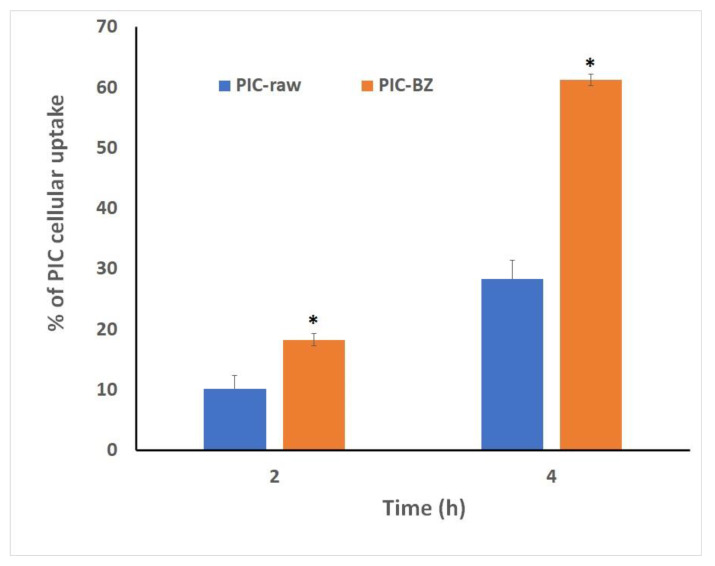
Cellular uptake of PIC from PIC raw and optimized PIC-BZ at 2 and 4 h. Data represent mean of six independent replicates SD. * Significantly different (*p* < 0.05) determined by Student’s *t*-test.

The quantitative cellular uptake of PIC by the PIC-BZ cells was assessed. The results show that the cellular uptake of the raw PIC was 10.2 ± 2.2% and 28.3 ± 3.2% at 2 and 4 h after starting the incubation, respectively. A higher uptake was observed with the optimized PIC-BZ incubations, which reached 18.32 ± 3.4% and 61.3 ± 5.4% at after 2 and 4 h of incubation, respectively (Figure 6).

#### 3.2.5. Cell Cycle Analysis

**Figure 7:**
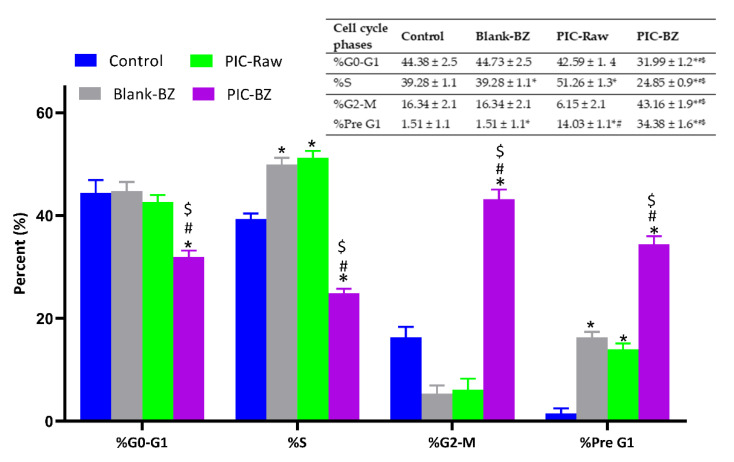
Impact of Blank-BZ, PIC-raw, or PIC-BZ treatments on A549 cell cycle phases with data table (inset). Data are the mean of 4 independent experiments ± SD. * Significantly different from control, *p* < 0.05; # significantly different from Blank-BZ, *p* < 0.05; $ significantly different from PIC-raw, *p* < 0.05.

As clearly shown in Figure 7, the optimized formulation (PIC-BZ) was able to significantly inhibit the proliferation of A549 cells compared to all the other treatments (*p* < 0.05 compared to all), with significant and very relevant changes (higher percentage of cells) occurring on G2/M and pre-G1 phases, accompanied by a significant reduction of cells in G0/G1 and S phases. Cell cycle analysis results revealed that blank-BZ showed non-significant effect when compared with PIC-raw at all phases of the cell cycle.

#### 3.2.6. Annexin V Staining

**Figure 8:**
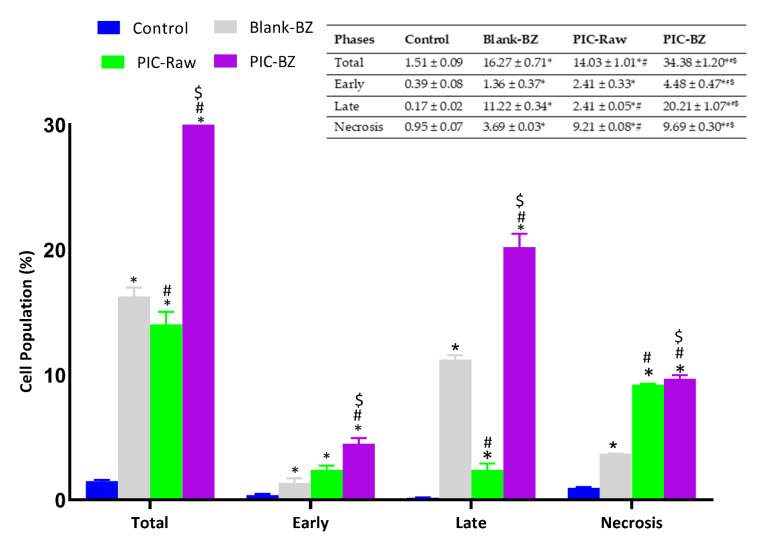
Impact of Blank-BZ, PIC-raw, or PIC-BZ treatments on the percentage of apoptotic or necrotic A549 cells with data table (inset). Total = apoptosis + necrosis; early = early apoptotic phase; late = late apoptotic phase. Data are the mean of 4 independent experiments ± SD. * Significantly different from control, *p* < 0.05; # significantly different from Blank-BZ, *p* < 0.05; $ significantly different from PIC-raw, *p* < 0.05.

With the aim to better understand whether the potentiated anti-proliferative effect of PIC-BZ treatment was also combined with pro-apoptotic and/or pro-necrotic activities, we investigated the impact of the different treatments on the percentage of apoptotic or necrotic A549 cells. As expected, on the basis of our previous findings, we found that the treatment of A549 cells with PIC-BZ was able to significantly increase the percentage of cell population in early and late stages of apoptosis, in necrosis, and in apoptosis + necrosis (indicated as total in the Figure 8) compared to control, Blank-PZ, and PIC-raw (*p* < 0.05), underlying the enhanced pro-apoptotic activity of the optimized formulation (Figure 8).

#### 3.2.7. Caspase-3 Content

**Figure 9:**
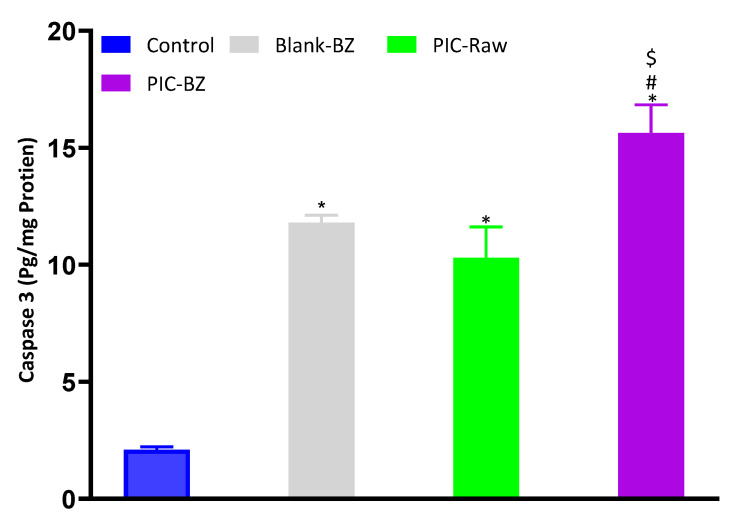
Effect of Blank-BZ, PIC-raw, or PIC-BZ treatments on caspase-3 enzyme contents in A549 cells. Values are expressed as pg/mg of protein. Data are the mean of 4 independent experiments ± SD. * Significantly different from control, *p* < 0.05; # significantly different from Blank-BZ, *p* < 0.05; $ significantly different from PIC-raw, *p* < 0.05.

Figure 9 shows that treatment of A549 cells with Blank-BZ or PIC-raw induced a significant increase of caspase-3 compared to control cells (*p* < 0.05). As expected, the maximal enhancement in caspase-3 content was observed in the case of PIC-BZ treatment (*p* < 0.05 compared to all the other experimental conditions).

#### 3.2.8. Wound Scratch Assay

**Figure 10:**
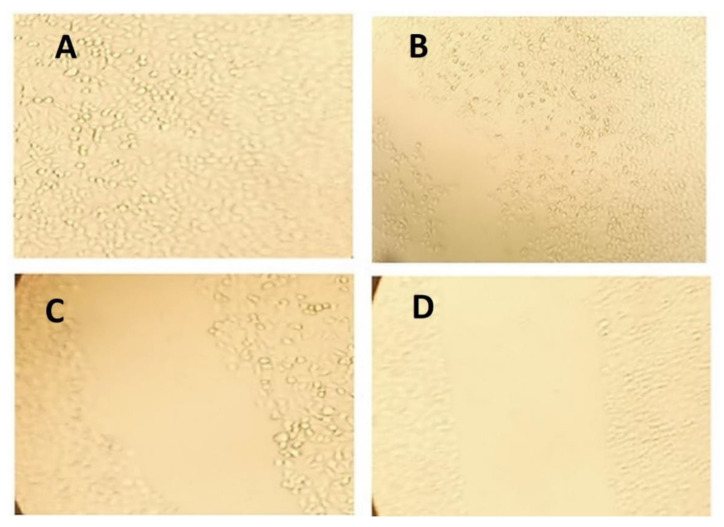
Wound coverage results of (**A**) untreated cells, (**B**) cells treated with blank-BZ, (**C**) cells treated with PIC-raw, and (**D**) cells treated with optimized PIC-BZ with 40× magnification.

The PIC-raw and PIC-BZ formulation demonstrated a drop in cell motility relative to untreated control (100% migration) and blank-BZ in the wound scratch assay. The wound coverages in the cases of Blank-BZ, PIC-raw, and PIC-BZ were found to be 77.03, 59.64, and 35.87%, respectively (Figure 10).

## 4. Discussion

Regarding model selection, the close agreement between the predicted and the adjusted R^2^ and the adequate precision value greater than four verified the applicability of the quadratic model for analyzing and navigating the experimental design space. The computed lack of fit F-value indicated no significance in relation to the pure error. There was a probability of 22.59%, and this value could be so high owing to noise. Non-significance lack of fit is recommended as the data should fit the selected model. In the diagnostic plots, the current λ represented by the blue line (Figure 1) was included in the computed confidence interval, indicating the fact that no specific data transformation was needed [54]. The waiver for transformation requirement was reinforced by the maximum to minimum measured particle size ratio of less than 10 (calculated ratio = 2.886). The random distribution of the points within the limits in both the externally studentized residuals vs. predicted response and the residual vs. run plots indicated that neither constant error nor lurking variable that can influence the measured particle size existed. Additionally, the highly linear pattern displayed in the predicted versus actual globule size plot reflected good coincidence between the observed and anticipated values [55,56]. Regarding the measured particle size, the comparatively low calculated standard deviation indicated homogenous and uniform distribution of the formulated bilosomal dispersions. The cholesterol/span ratio was found to be the most significant factor influencing the particle size, as evidenced by its highest coefficient in the polynomial equation as compared to the other factors (Figure 2). The observed decrease in particle size with increasing cholesterol/span ratio could be credited to decreased relative concentration of cholesterol at higher ratios. It has been previously reported that increased levels of cholesterol impedes adjacent packing of the vesicles lipids, resulting in high membrane fluidity that in turn leads to enhanced distribution of aqueous phase within the vesicle, with subsequent increase in particle size [29,57].

We showed that PIC is well entrapped within the optimized PIC-BZ formula and it could be attributed to the ability of bilosomes as well as to the hydrophobic nature of zein to encapsulate PIC [29,44,58]. The release could be considered well-enhanced, considering the poor solubility of the PIC. In contrast to our findings, the in vitro release of PIC encapsulated in albumin nanoparticles was found to be very low [11]. For the different PIC-loaded formulations, albumin nanoparticles may release about 20–40%. In this study, it may be clarified that the solubilizing power of BZ against the hydrophobic drug depended on the virtue of the lipophilic nature of its components. The ability of BZ to improve the solubility poorly water-soluble drugs has been previously stated [59].

As compared to PIC-raw, PIC-BZ exhibited a fourfold decrease in the IC_50_ value (Figure 5). This result is in accordance to a previous study that demonstrated PIC cytotoxic activity in A549 cells [60]. In A549 lung cancer cells, the cytostatic activity of PIC-BZ has been confirmed. It may be inferred that the PIC cytotoxicity was greatly enhanced by its formulation of BZ. A reason for the enhanced cytotoxicity may be given by high cellular permeability. On the basis of the lipophilic nature of the delivery system along with the nano-size range, one can understand the indicated high permeability [61,62]. In addition, optimized PIC-BZ showed improved cellular PIC uptake compared to raw PIC (Figure 6). This indicates the ability of the optimized BZ formula to improve the delivery of PIC across cellular barriers. Assessing the influence of PIC-BZ on cell cycle phases showed aggregation of cells in both G2-M and pre-G1 phases (Figure 7). The findings are supported by a previous PIC study, which highlighted the PIC’s potential to induce G2-M phase in SK-Mel-28 melanoma cells, an effect due to the downregulation of cyclins A, E, and B1. Furthermore, the percentage of apoptotic cells in the pre-G phase was significantly increased by PIC [63]. However, recorded studies have also shown that in the G0-G1 phase, PIC blocks cells [64]. The relatively low concentrations used in the present study can explain this. The observed increased fraction of A549 cells with positive annexin staining (Figure 8) highlighted improved pro-apoptotic activity. This is in line with previous studies that proposed apoptosis induction as a mechanism of PIC antiproliferative properties [65]. In this regard, the significantly enhanced activity of PIC-BZ related to pure PIC is remarkable. This confirms the role of BZ in enhancing pro-apoptotic PIC activities. Furthermore, PIC-BZ demonstrated a significant rise in the proportion of A549 cells with positive annexin staining. This is in line with other works demonstrating BZ’s potential to improve annexin V staining of A549 cells when loaded with piperine and curcumin [66]. Such findings may be attributed to the lipophilic nature of the formulation that provides enhanced antiproliferative agent delivery [67]. It must be indicated that blank BZ demonstrated a significant proliferation inhibitory activity. This could be attributed to the cytotoxic effect of zein (component of blank formula) that has been previously reported to show similar effects [68]. The blank BZ formula enhanced the pre-G phase, indicating apoptotic cell death. Effect of blank BZ formula was further confirmed by their effect on annexin V staining, which showed significant apoptotic-enhancing activity through early late and total cell death (Figure 8). These results were in accordance with previous reports [58,68,69]. In the present analysis, caspase-3 content was significantly increased by PIC-BZ compared to all other conditions (Figure 9). This is in line with the previous studies demonstrating PIC’s potential to increase cellular caspase-3 [70,71]. Furthermore, the improved results of BZ on the cleaved caspase-3 material of A549 cells have been previously shown [72]. The last cytosolic occurrence preceding apoptosis is the elevation of the cleaved caspase-3 material. It can also be deduced that the formulating of PIC in a nanostructured system substantially increases the content of caspase-3. The enhanced anti-proliferative activity of the optimized formula against cancer cells was also corroborated by the results of the scratch assay (Figure 10).

## 5. Conclusions

In this study, in the PIC-BZ formulation and optimization, the Box–Behnken design was successfully implemented and used. The prepared BZ demonstrated nano-size (157.27 nm) and high drug entrapment. In addition, the preparation of the optimized BZ with decreased particle size and maximized drug trapping demonstrated a gradual and complete in vitro release spherical form. The in vitro experiments carried out on A549 cells clearly demonstrated as the optimized formula (PIC-BZ) significantly improved all the parameters related to the cytotoxic potential towards cancer cells, including the decrease of IC_50_, the enhancement of anti-proliferative activity, the increase of apoptosis and necrosis cell populations paralleled by an increment of intracellular caspase-3 concentration, and inhibition of wound closure.

## Figures and Tables

**Table 1:** Formulation variables’ levels and particle size constraint used in the Box–Behnken design for the formulation and optimization of PIC-BZ.

Independent Variables	Levels		
	(−1)	(0)	(+1)
X_1_: Cholesterol/span ratio	1:2	1:3	1:4
X_2_: Bile salt molar concentration (mM)	0.250	0.375	0.500
X_3_: Zein concentration (%*w*/*w*)	5.0	7.5	10.0
Responses	Desirability Constraints		
Y_1_: Particle size (nm)	Minimize		

Abbreviations: PIC-BZ, piceatannol-loaded bilosome–zein.

**Table 2:** Variables levels and observed particle size for PIC-BZ experimental runs prepared according to Box–Behnken design.

Experimental Run #	Independent Variables			Particle Size (nm) ± SD
	Cholesterol/Span Molar Ratio	Bile Salt Concentration (mM)	Zein Concentration (%*w*/*w*)	
F1	1:2	0.375	10.0	459.2 ± 16.3
F2	1:3	0.375	7.5	215.8 ± 7.6
F3	1:4	0.375	5.0	173.4 ± 5.9
F4	1:4	0.250	7.5	239.2 ± 8.1
F5	1:4	0.375	10.0	280.3 ± 9.8
F6	1:3	0.375	7.5	226.5 ± 4.2
F7	1:3	0.250	5.0	274.6 ± 6.9
F8	1:2	0.375	5.0	296.2 ± 12.8
F9	1:2	0.250	7.5	397.8 ± 13.1
F10	1:3	0.250	10.0	411.1 ± 12.9
F11	1:3	0.375	7.5	221.1 ± 6.5
F12	1:3	0.500	5.0	189.3 ± 3.9
F13	1:3	0.375	7.5	216.9 ± 7.9
F14	1:3	0.500	10.0	297.8 ± 13.6
F15	1:2	0.500	7.5	285.3 ± 9.8
F16	1:3	0.375	7.5	218.9 ± 5.8
F17	1:4	0.500	7.5	159.6 ± 4.6

Abbreviations: PIC-BZ, piceatannol-loaded bilosome–zein; SD, standard deviation.

**Table 3:** Model summary statistics for selection of the best fitting model for the particle size of PIC-BZ.

Source	SD	R^2^	Adjusted R^2^	Predicted R^2^	PRESS
Linear	39.52	0.8241	0.7835	0.7310	31,055.92
2FI	43.65	0.8350	0.7360	0.5643	50,299.07
Quadratic	5.24	0.9983	0.9962	0.9823	2042.22

Abbreviations: PIC-BZ, piceatannol-loaded bilosome–zein; SD, standard deviation; PRESS, predicted residual error sum of squares; 2FI, two-factor interaction.

## Data Availability

Data are included in the manuscript.
